# Yield stability and economic heterosis analysis in newly bred sunflower hybrids throughout diverse agro-ecological zones

**DOI:** 10.1186/s12870-022-03983-1

**Published:** 2022-12-12

**Authors:** Nihar Ranjan Chakraborty, Shyam Sundar Lakshman, Sandip Debnath, Mehdi Rahimi

**Affiliations:** 1 Department of Genetics and Plant Breeding, Institute of Agriculture, Visva-Bharati University, Sriniketan, 731236 Santiniketan, West Bengal India; 2 AICRP Sunflower, Nimpith Centre, South 24 Parganas, Nimpith,, West Bengal 743338 India; 3 Department of Biotechnology, Institute of Science and High Technology and Environmental Sciences, Graduate University of Advanced Technology, Kerman, Iran

**Keywords:** Sunflower, Hybrids, Multi-location trials, G × E interaction, Seed and oil yield, Stability, Economic heterosis

## Abstract

Exploration of heterosis is a strategy for enhancing sunflower yield and productivity. In India, the greatest constraints on sunflower production are stagnant and inconsistent yields. By raising them in a variety of ecological conditions, stable *per-se* performance with the highest yielding potential sunflower hybrids were selected. Sustainable agriculture requires the use of desirable hybrids with high seed yields and oil content too. By making three distinct crossing sets from 32 sunflower genotypes, 11 cytoplasmic male sterility (CMS), and 21 restorer lines, a total of 124 hybrids were developed (comprising both lines and tester). After extensive field evaluation of all hybrids, only eight superior F_1_s belonging to all three sets, as well as the three national control hybrids KBSH-53, LSFH-171, and DRSH-1, were selected for stability analysis in four agro-ecological regions of West Bengal, India viz., Nimpith, Baruipur, Bankura, and Berhapore. The genetic stability of several phenotypic characters was assessed using statistical models that examine genotype-environment interaction (G × E) in multi-locational yield trials. In this experiment, the performance of hybrids under various environmental circumstances over two-year periods was measured using regression coefficient (b_i_) and deviations from regression (S^2^d_i_). With the exception of genotypes CMS-852A × EC-601751 for volume weight (0.9335) and CMS-302A × EC-623011 for head diameter (0.0905) and volume weight (0.6425), all sunflower genotypes for all concerned traits had extremely minor and negligible deviations from regression (S^2^d_i_), which showed significant values. The genotypes having insignificant values of S^2^d_i_ were more stable. The economic heterosis of these novel hybrids was also quantified. CMS-302A × EC-623011 in which seed yield was recorded 20.90, 20.91, 20.95 and 20.90% higher than DRSH-1 at Nimpith, Baruipur, Bankura and PORS (Berhampur), respectively. The research revealed that CMS-302A × EC-623011, CMS-853A × EC-623027 and P-2–7-1A × EC-512682 exhibited good seed production and stability for critical agronomic parameters in addition to oil content. As a result, the current researches enlighten to find out how stable the expression of important economic traits in sunflower hybrids is.

**Supplementary Information:**

The online version contains supplementary material available at 10.1186/s12870-022-03983-1.

## Introduction

Sunflower (*Helianthus annuus* L.) is the world’s thrid most important source of vegetable oil [1] due to its low to moderate production requirements, high oil quality, protein content, and utilisation of all plant parts. It is also considered a good quality oil due to its high concentration of polyunsaturated fatty acids. It's also a photo-insensitive crop that gets a lot of cross-pollination from the protandrous stamens and honey bee pollination. Population breeding was moved to heterosis breeding after the discovery of cytoplasmic male sterile (CMS) lines [2, 3] and fertility restorer lines [4, 5]. In the 1970s, commercial sunflower cultivation began in India with the screening of imported open-pollinated populations. Stagnant and uneven yields, as well as vulnerability to a number of biotic stresses throughout the crop's life cycle, are the primary restrictions on sunflower production in India [6]. Because the tiny genetic base is a key bottleneck for breaking the yield plateau, it is vital to boost sunflower seed and oil yield by integrating additional genes from superior lines through recombination breeding. Plant breeders are very interested in genetic variety [7–9]. The more different the parents, the more likely a heterotic cross combination will result in F_1_ with a wide range of diversity in segregating generations [10]. The various CMS and restorer lines are intended to contribute to the generation of superior hybrids with high seed yield and oil content, as well as improved heterosis and stability [11, 12]. Heterosis exploration is an approach to increasing sunflower yield and productivity. Sunflower production has been rapidly superseded by high-yielding hybrids, which were first dominated by open-pollinated varieties. However, due to poor seed filling, low yield levels, low oil content, and vulnerability to disease and insects, many hybrids failed to catch the agricultural community's interest as valuable crops. These occurrences could happen as a result of the G × E interaction [13]. As a result, it was decided that high heterotic stable crosses should be sought after. Before selecting acceptable genotypes, a large number of candidate genotypes are frequently tested in a range of situations for crucial yield-related traits. Location, seasonal fluctuation, and their combination had a substantial impact on genotype performance in terms of yield and yield contributing potential. One of the essential properties of a genotype that permits it to be released as a cultivar with a wide range of uses is its performance stability [14]. To develop the necessary criteria for ranking genotypes for stability, a variety of methods are available, including stability variance, coefficient of regression, and mean squared deviations from regression [15–17]; fixed coefficient of regression [18]; and others with acceptable parameters. There is a scarcity of data on genotype-environment interaction and hybrid stability in sunflowers. The goal of this study was to investigate the stability of yield-related characteristics in promising sunflower hybrids and to estimate economic heterosis (%) for seed output and oil yield over National Checks (hybrids) under different situations.

## Material and methods

### Experimental materials

This study complied with relevant institutional, national, and international guidelines and legislation of India, and no specific permits were required to collect the plant materials. So, the 32 sunflower genotypes (11 CMS lines and 21 restorer lines) were obtained from the Oilseeds Research Station in India and were used for study to investigate their heterogeneous origin, growth habit, phenology, and adaption (Supplementary files Table 1).

**Table 1:** Analysis of variance of sunflower hybrids for stability in seven yield attributing traits

Hybrid	df	Seedyield (kg/ha)	Oil content (%)	Oil yield (kg/ha)	100 seed weight (g)	Plant height (cm)	Head diameter (cm)	Volume weight (g/100 cc)
Genotypes (G)	10	57,944.2**	14.24**	10,798.820**	1.317**	643.00**	0.874**	8.710**
E + (G × E)	33	11,589.4**	0.887**	2930.570**	0.015**	43.58**	0.069*	1.189**
Environments (E)	3	126,768.2**	9.73**	32,021.670**	0.139**	411.83**	0.481**	8.559**
G × E	30	71.66*	0.003**	21.460**	0.003	6.76	0.028	0.451
Environments (Linear)	1	380,304**	29.20**	96,065.020**	0.416**	1345.41**	1.442**	25.67**
G × E (Linear)	10	163.4**	0.006**	54.610**	0.001	10.93	0.012	0.575
Pooled deviation	22	23.41	0.001	4.441	0.003	4.25	0.032*	0.354
Pooled error	40	1007.7	0.073	176.347	0.020	4.68	0.017	0.149
Total	43	22,369.2	3.393	4760.396	0.318	182.98	0.256	2.938
S.E.D. Genotype. Gi—Gj		3.4216	0.0166	1.4901	0.0391	1.4590	0.1272	0.4209
S.E.D. Environ. Ei—Ej		2.0633	0.0100	0.8986	0.0236	0.8798	0.0767	0.2538
LSD (at *P* = 0.05)(Geno.)		7.0960	0.0344	3.0902	0.0811	3.0258	0.2637	0.8729
LSD (at *P* = 0.05)(Env.)		4.2791	0.0207	1.8635	0.0489	1.8246	0.1590	0.5264

*S.E.D* Standard error of a difference, *LSD* Least Significant Difference

^*, **^, Significant at *p* = 0.05 and 0.01 respectively

### Experimental site

The experiment was conducted at four diverse agro-ecological zonesin West Bengal namely: the RAKVK Research Farm’s Nimpith, Baruipur, Bankura and PORS (Berhampur). Nimpith is located at latitude of 22°33’ north and a longitude of 89°4’ east, at an elevation of 1.5 m above mean sea level. Baruipur is altitude of 22°21’ north and a longitude of 88°25’ east. Bankura is situated between of 22°38’ and 23°38’ north latitude and between 88°36’ and 87°46’ east longitude. PORS (Berhampur): latitude 24°5’ north and a longitude 88°16’ est.

#### Weather data of experimental area

The weather data of the experimental area during 2017–2018 and 2018–2019 are depicted in Supplementary files (Tables 2 and 3). Data showed that the temperature average (High/low), Humidity average and rainy day which was recorded during December to April.

**Table 2:** Performance of sunflower hybrids in seven yield attributing traits at four different locations

Hybrids	Location	Yield attributing traits						
		Seed yield (kg/ha)	Oil content(%)	Oil yield (kg/ha)	100 seed weight (g)	Plant height (cm)	Head diameter (cm)	Volume weight (g/100 cc)
CMS-853A × EC-623027	Nimpith	2310	37.4	863.94	6.1	170	15.7	39.35
	Baruipur	2113.5	36.3	767.20	5.95	160	15.4	38.15
	Bankura	2394.5	38.1	912.30	6.2	166.5	15.8	40.05
	Berhampur	2229.5	38.5	858.36	6.15	173.5	15.5	39.1
	Average	2261.88	37.58	850.45	6.1	167.5	15.6	39.16
P-2–7-1A × EC-601958	Nimpith	2251.5	37.6	846.56	6.15	156.5	14.6	40.75
	Baruipur	2049.5	36.4	746.02	6	155	14.4	39.5
	Bankura	2310.5	38.2	882.61	6.3	160.5	15.1	40.75
	Berhampur	2162	38.6	834.53	6.2	167	15.1	41.5
	Average	2193.38	37.7	827.43	6.16	159.75	14.8	40.63
P-2–7-1A × EC-512682	Nimpith	2299.5	37.7	866.91	5.95	162	16.2	39.9
	Baruipur	2104	36.6	770.06	5.85	158	16	38.6
	Bankura	2371	38.4	910.46	6.1	165.5	16.3	41.5
	Berhampur	2220	38.8	861.36	6.05	173.5	16.1	40.6
	Average	2248.63	37.88	852.20	5.99	164.75	16.15	40.15
CMS-302A × R-12–96	Nimpith	2191	35.2	771.23	5.05	160.5	15.4	38.1
	Baruipur	2005	34.15	684.71	4.9	159	14.7	38.15
	Bankura	2242.5	35.85	803.94	5.15	164.5	15.1	40.35
	Berhampur	2115.5	36.25	766.87	5.1	166.5	15.2	39.95
	Average	2138.25	35.36	756.69	5.05	162.63	15.1	39.14
CMS-852A × EC-601751	Nimpith	2094.5	34.55	723.65	5.4	154	15.8	36.1
	Baruipur	1916.5	33.55	642.99	5.25	150	15.5	36.5
	Bankura	2151.5	35.25	758.40	5.5	160	15.9	38.2
	Berhampur	2021.5	35.6	719.65	5.5	162.5	16	37.25
	Average	2046	34.74	711.17	5.41	156.63	15.8	37.01
CMS-302A × EC-512682	Nimpith	2170	36.95	801.82	5.85	174	14.7	38.5
	Baruipur	1985.5	35.85	711.80	5.7	166.5	14.4	37.15
	Bankura	2224	37.65	837.34	5.95	170.5	15	40.3
	Berhampur	2094.5	38.05	796.96	5.9	175.5	15	41.95
	Average	2118.5	37.13	786.98	5.85	171.63	14.78	39.48
CMS-852A × EC-623023	Nimpith	2240.5	37.25	834.59	5.4	157.5	15.3	37.3
	Baruipur	2050	36.15	741.08	5.25	155	15	36.65
	Bankura	2296	37.95	871.33	5.5	163	15.4	37.75
	Berhampur	2163	38.35	829.51	5.65	169	15	38.65
	Average	2187.38	37.43	819.13	5.45	161.15	15.18	37.59
CMS-302A × EC-623011	Nimpith	2360.5	36.4	859.22	5.7	159	15.3	40.75
	Baruipur	2160	35.3	762.48	5.55	157.5	14.7	39.75
	Bankura	2419	37.1	897.45	5.8	163.5	15	39.35
	Berhampur	2279	37.5	854.63	5.6	174	14.6	41.5
	Average	2304.62	36.58	843.44	5.66	163.5	14.9	40.34
LSFH-171 (NC 1)	Nimpith	2381	32.3	769.06	5.85	194	15.9	35.75
	Baruipur	2179	31.3	682.03	5.7	185	15.4	35.85
	Bankura	2422.5	32.9	797.00	5.95	200	16	36.45
	Berhampur	2298.5	33.2	763.10	5.85	206	15.8	37.45
	Average	2320.25	32.43	752.80	5.84	196.25	15.78	36.38
DRSH-1 (NC 2)	Nimpith	1952.5	38.7	755.62	6	174.5	15.2	40
	Baruipur	1786.5	37.5	669.94	5.85	166	14.7	39.7
	Bankura	2000	39.4	788.00	6.1	179.5	15.4	40.65
	Berhampur	1885	39.8	750.23	6.1	184.5	15.1	41.5
	Average	1906	38.85	740.95	6.01	176.13	15.2	40.46
KBSH-53(NC 3)	Nimpith	2217	34.45	763.76	4.15	187.5	15.6	37.75
	Baruipur	2029	33.45	678.70	4.05	178.5	15.3	37.25
	Bankura	2277.5	35.15	800.54	4.25	193.5	16	39.45
	Berhampur	2141	35.5	760.06	4.4	199	15.7	38.7
	Average	2166.13	34.64	750.76	4.21	189.63	15.65	38.29
Mean	Nimpith	2224.36	36.23	805.12	5.60	168.14	15.43	38.57
	Baruipur	2034.41	35.14	714.27	5.46	162.77	15.05	37.93
	Bankura	2282.64	36.90	841.76	5.71	171.55	15.55	39.53
	Berhampur	2146.32	37.29	799.57	5.68	177.36	15.37	39.83
	Average	2171.93	36.39	790.18	5.61	169.95	15.35	38.96
C. V	Nimpith	5.54	5.21	6.34	10.50	7.87	3.15	4.53
	Baruipur	5.54	5.19	6.29	10.60	6.51	3.41	3.59
	Bankura	5.57	5.16	6.54	10.43	7.95	3.02	3.81
	Berhampur	5.54	5.18	6.28	9.47	7.77	3.11	4.27
SE of difference	Nimpith	37.17	0.57	15.39	0.18	3.99	0.15	0.53
	Baruipur	33.96	0.55	13.55	0.17	3.19	0.15	0.41
	Bankura	38.33	0.57	16.61	0.18	4.11	0.14	0.45
	Berhampur	35.82	0.58	15.14	0.16	4.16	0.14	0.51
LSD 0.05	Nimpith	82.81	1.27	34.29	0.40	8.89	0.33	1.17
	Baruipur	75.63	1.23	30.19	0.39	7.12	0.34	0.91
	Bankura	85.41	1.28	37.00	0.40	9.16	0.32	1.01
	Berhampur	79.88	1.30	33.73	0.36	9.26	0.32	1.14

**Table 3:** Mean values of yield attributing traits over locations with corresponding S^2^d_i_ and b_i_ of the sunflower hybrids

Hybrids	Seed yield (kg/ha)			Oil content (%)			Oil yield (kg/ha)			100 seed weight (g)			Plant height (cm)			Head diameter (cm)			Volume weight (g/100 cc)		
	Mean	S^2^d_i_	b_i_	Mean	S^2^d_i_	b_i_	Mean	S^2^d_i_	b_i_	Mean	S^2^d_i_	b_i_	Mean	S^2^d_i_	b_i_	Mean	S^2^d_i_	b_i_	Mean	S^2^d_i_	b_i_
CMS-853A × EC-623027	2261.87	149.91	1.137**	37.57	0.00023	1.022**	850.4	24.15	1.135**	6.10	0.00023	1.022**	167.5	9.749	0.812	15.63	0.00349	0.871*	39.16	0.4083	0.617
P-2–7-1A × EC-601958	2193.37	561.34	0.979**	37.70	0.1422	0.923**	827.5	28.01	1.085**	6.16	0.14224	0.923**	157.7	6.689	0.775	14.78	0.06113	1.051**	40.62	0.2696	0.879*
P-2–7-1A × EC-512682	2248.62	67.717	1.049**	37.87	0.0545	0.964**	852.2	10.19	1.107**	5.98	0.0545	0.964**	164.8	9.327	0.999**	16.12	0.01159	0.666	40.15	0.3754	1.248**
CMS-302A × R-12–96	2138.50	66.35	0.988**	35.36	0.1638	0.871*	756.8	5.048	0.956**	5.05	0.16379	0.871*	162.6	3.052	0.574	15.06	0.03945	1.117**	39.13	0.2875	1.231**
CMS-852A × EC-601751	2046.00	106.05	0.571	34.73	0.0359	1.01**	711.4	118.47	0.897*	5.41	0.03589	1.009**	156.6	3.197	0.934**	15.78	0.02248	0.866*	37.01	0.9335	0.779
CMS-302A × EC-512682	2118.50	228.14	1.136**	37.12	0.5168	1.209**	786.3	6.018	1.004**	5.85	0.5168	1.209**	171.6	14.297	0.631	14.73	0.02337	1.216**	39.47	0.5743	1.211**
CMS-852A × EC-623023	2187.37	644.66	1.085**	36.57	0.000227	1.022**	800.4	9.249	1.026**	5.45	0.000227	1.022**	161.1	7.505	0.932	15.17	0.05405	1.126**	37.58	0.0996	0.922**
CMS-302A × EC-623011	2304.62	7.757	1.051**	37.42	.00231	1.037**	862.9	7.094	1.097**	5.63	0.00231	1.037**	163.5	17.739	1.056**	14.86	0.09052	0.648	40.44	0.6425	0.529
LSFH-171 (NC 1)	2466.25	830.91	1.096**	32.42	0.00097	0.896*	752.7	166.42	0.776	5.83	0.000975	0.896*	196.2	4.245	1.478**	15.75	0.09909	1.099**	36.37	0.5337	0.653
DRSH-1 (NC 2)	1906.00	58.48	0.835*	38.85	0.00079	1.066**	740.9	5.806	0.945**	6.01	0.00079	1.066**	176.1	11.838	1.373**	15.08	0.03696	0.962**	40.46	0.3749	0.939*
KBSH-53(NC 3)	2166.12	455.37	1.073**	34.63	0.0065	0.980**	750.6	17.556	0.971**	4.21	0.00651	0.980**	189.6	3.230	1.436**	15.63	0.02347	1.381**	38.29	0.2640	0.988**
GM	2171.93			36.39			790.18			5.61			168.95			15.32			38.97		
SEm ( ±)	2.80			0.014			1.20			0.032			1.20			0.103			0.343		

^*,^^**^ Significant at *p* = 0.05 and 0.01, respectively

#### Soil of the experimental location

The field's topsoil was removed prior to the application of basal fertilisers. The experimental plot's soil is a sandy loam with a medium to low fertility status and is acidic, resembling more or less red and lateritic soils except PORS (Berhampur) where soil is with a pH of 6.5, organic matter (carbon percent) 0.5, available N (kg/ha) 235.4, available P (kg/ha) 20.4, and available K (kg/ha) 175.5.

### Field techniques

Three distinct crossing sets (comprising both lines and tester) were produced from the 32 genotypes. The hybridization programme was conducted in 2015 and 2016 at the AICRP-Sunflower, Nimpithcentre, during the Kharif season. Cytoplasmic male sterile (CMS) A-lines were maintained by crossing with their respective maintainer (B) lines, whereas maintainer (B) lines were maintained by selfing. On the basis of flowering synchronisation, hybridization was initiated between the parents at the outset of flowering. CMS lines were employed in this hybridization programme. The CMS lines were raised in one block and the restorer lines in the next block in the crossing block. The pollination of chosen CMS flowers was accomplished by collecting pollen from previously bagged heads before blossoming. Bags were placed over male and female flowers a day prior to prevent contamination and to avoid spilling pollen. Pollen was collected into paper bags from chosen flowering heads using a light tap of the hand on the back of the head. Pollen grains were administered in the morning between 9 and 11 AM using a camel hairbrush dipped in pollen and gently dragged over the receptive surface of the stigmas. Pollination was performed in each combination for five to six days (on alternate days) to guarantee an adequate seed set. Following pollination, flowers were packaged and properly labelled for storage until harvesting.

- SET I: 36 F1/hybrids were developed using 4 CMS lines and 9 testers.
- SET II: 32 F1/hybrids were developed using 4 CMS lines and 8 testers.
- SET III: 56 F1/hybrids were developed using 8 CMS lines and 7 testers.

Field evaluations were conducted in 2015–16 and 2016–17 at AICRP-Sunflower, Nimpith, during the *Rabi* season. All three sets of F1s (newly developed hybrids) and their parents were evaluated in a randomised complete block design with three replications in a plot size of 1.8 m × 3.0 m with a row spacing of 60 cm and a plant to plant spacing of 30 cmalongside the three national check hybrids, KBSH-53, LSFH-171, and DRSH-1.

Stability analysis was conducted on eight superior sunflower hybrids belonging to all three sets CMS-853A × EC-623027, P-2–7-1A × EC-601958, P-2–7-1A × EC-512682, CMS-302A × R-12–96, CMS-852A × EC-601751, CMS-302A × EC-512682, CMS-852A × EC-623023, and CMS-302A × EC-623011, as well as three national check hybrids KBSH-53,LSFH-171, and DRSH-1 The studies were done in a randomised complete block design with three replications on a plot size of 4.5 m × 3.0 m in West Bengal during the *Rabi* season of 2017–18 and 2018–19. Each plot contained five rows with a row spacing of 60 cm and a plant to plant spacing of 30 cm. Standard agronomic packages and techniques were used during the experimental periods to maintain a healthy crop.

### Characters studied

The observations for all the traits were recorded on 10 randomly selected competitive plants for each genotype in each replication in each environment. A brief description of the procedure adopted for recording the observations of various traits was as under:

- Plant height (cm): The height of fully matured plant from the base of the plant to the basal surface of the capitulum was recorded as plant height.
- Head diameter (cm): The head diameter was recorded from both the diagonal axes at maturity.
- 100 seed weight (g): Seeds of sample plants from each entry were bulked, dried and cleaned. Three samples containing 100 seeds were drawn from each lot and 100-seed weight was recorded as average of those 3 lots in gram (g) using semi-micro electronic balance.
- Volume weight (g/100 ml): The measuring cylinder was filled to 100 ml volume with seeds of each entry and was weighed in gram as volume weight/100 ml using semi-micro electronic balance.
- Seed yield (kg/ha): Seed yield per plant (g) converted to seed yield (kg/ha) by multiplying with the conversion factor (55.55). (10000m^2^/0.60 × 0.30) × 1000(kg/ha).
- Oil content (%): A randomly bulk sample of filled seeds was drawn from selected plant produce weighing 50 g from each entry in each replication. The oil content in percentage was measured using Nuclear Magnetic Resonance (NMR) facility available at Institute of agriculture, PSB, Visva-Bharati University, Sriniketan and Bidhan Chandra Krishi Viswa Vidyalaya, Mohanpur, Nadia, West Bengal.
- Oil yield (kg/ha): Seed yield (kg/ha) multiply by oil content (%).

### Statistical methods

The phenotypic stability of a genotype was assessed using the Eberhart and Russell [15] model. The statistical model of the analysis was as follows:

$${\mathrm{Y}}_{\mathrm{ij}}={\mathrm{u}}_{\mathrm{i}}+{\upbeta }_{\mathrm{i}}{\mathrm{I}}_{\mathrm{j}}+{\updelta }_{\mathrm{ij}}$$

where,

Y_ij_ = mean performance of i^th^ genotype in j^th^ environment.

μ_i_ = mean of i^th^ genotype over all the environments.

β_i_ = the regression coefficient of i^th^ genotype.

δ_ij_ = deviation from regression of the i^th^ genotype.

I_j_  =  the environmental index for j^th^ environment.

### Estimation of stability parameters

Two parameters of stability viz*.* regression coefficient (b_i_) and deviations from regression (S^2^d_i_) were calculated.

I. The regression coefficient (b_i_) is the regression of the performance of each genotype under different environments on the environmental means over all the genotypes. It was estimated as:

 

$${b}_{i}= \frac{\sum_{j=1}^{l}{Y}_{ij}\times {I}_{J}}{\sum_{j=1}^{l}{I}_{J}^{2}}$$

II. The deviations from regression (S^2^d_i_) was estimated as:

 

$${S}_{di}^{2}=\left(\frac{\sum_{j=1}^{l}{\widehat{\delta }}_{ij}^{2}}{l-2}\right)-\frac{{S}_{e}^{2}}{r}$$

where,

S^2^_e_/r = estimate of pooled error mean square and

$$\sum_{j}^{l}{\widehat{\delta }}_{ij}^{2}=\left({\sum }_{j=1}^{i}{Y}_{ij}^{2}-\frac{{Y}_{i}^{2}}{l}\right)-\frac{\left(\sum_{j=1}^{i}{Y}_{ij}^{2}.{I}_{j}\right)}{\sum_{j=1}^{i}{I}_{j}^{2}}$$

Economic heterosis:

$$\frac{\left({\overline{F}}_{1}-\overline{BC}\right)}{\overline{BC}}\times 100$$

Its significance was tested by ‘t’ test as follows

$${t}_{EDF}=\frac{\left({\overline{F}}_{1}-\overline{BC}\right)}{SE\left({\overline{F}}_{1}-\overline{BC}\right)}\times 100$$

$$E\left({\overline{F}}_{1}-\overline{BC}\right)=\sqrt{\frac{2MSE}{n}}$$

where,

F_1_  =  mean value of hybrid.

BC  =  mean value of best genotype among checks and parents.

n = divisor in respective conditions i.e. r in case of individual environment and rs in over the environments

r,s  =  number of replications and environments, respectively.

MSE = error mean square from (Table 3.8 and 3.9 for individual and over the environments, respectively

t_EDF_  =  Students ‘t’ at error degrees of freedom.

To calculate economic heterosis parent and check had higher mean values were considered desirable for all the characters.

The data were analysed in the computer using the Windostat version 8.6 from Indostat service Hyderabad, India.

## Results

Analysis of variance (Table 1) indicated extremely substantial variations between genotypes and environments, indicating the presence of hybrids and environmental variation. For 100 seed weight, plant height, head diameter, and volume weight, there were no significant G × E interactions, showing that hybrids maintained a consistent response to environmental changes for those variables.

The occurrence of significant G × E interaction variation for the other traits, seed yield, oil content, and oil yield, indicates that hybrids respond differently under different environmental conditions. The regression study found that the mean sum of squares attributable to the environment (linear) was very significant for all attributes investigated, indicating that a considerable part of variance may be attributed to linear regression. The importance of G × E (linear) for seed yield, oil content, and oil yield indicates that variance in genotype performance is due to genotypes' regression to their environment, and hence performance is predicted. The mean squared deviation of G × E (linear) was not found significant to 100 seed weight, plant height, head diameter and vol. weight implying that unpredictable components of genotype-environment interaction had a greater impact. Langhi, Patel [19] reported similar non-significant variations owing to genotype × environment for plant height. Thus, both linear and non-linear components played a role in determining stability. For the majority of the characteristics, both the linear and non-linear components of G × E were significant, suggesting the importance of both regression and divergence from the regression in determining stability. These findings corroborate those of Bhoite, Mane [20].

### Performance of sunflower hybrids

The performance of sunflower hybrids in seven yield attributing traits at four different locations is shown in Table 2. In seed yield, the average mean value was 2171.9 kg/ha. Highest seed yield was recorded in the hybrid named CMS-302A × EC-623011 (2419.0 kg/ha) which was followed by CMS-853A × EC-623027 (2394.5 kg/ha), P-2–7-1A × EC-512682 (2371.0 kg/ha) and P-2–7-1A × EC-601958 (2310.5 kg/ha). All these highest yield was recorded at Bankura Centre where out-yielded national check sunflower hybrids such as DRSH-1 (2000 kg/ha) are statistically comparable to LSFH-171 (2422.5 kg/ha) and KBSH-53 (2277.5 kg/ha). The lowest yield was recorded at Baruipur in hybrids named CMS-852A × EC-601751, CMS-302A × EC-512682 having 1916. 5, 1985.5 kg/ha, respectively. In terms of oil content all the hybrids outperformed in terms of average oil content considering all the four centres over the two national check hybrids LSFH-171 (32.43%) and KBSH-53 (34.64%). The hybrids CMS-302A × EC-623011 (862.95 kg/ha), P-2–7-1A × EC-512682 (852.23 kg/ha), CMS-853A × EC-623027 (850.4 kg/ha), P-2–7-1A × EC-601958 ( 827.5) and CMS-852A × EC-623023 (800.43 kg/ha) yielded more oil than the grand mean (790.16 kg/ha) across all environments and out-yielded national check sunflower hybrids such as LSFH-171 (752.7 kg/ha), DRSH-1 (740.93 kg/ha) and KBSH-53 (750.63 kg/ha). Table 2 explains that the average mean value was 5.68 g for 100 seed weight considering all four environments. Higher number of weight in gram (6.16) was noticed in P-2–7-1A × EC-601958whichwas followed by the hybrid CMS-853A × EC-623027, P-2–7-1A × EC-512682, CMS-302A × EC-512682 and CMS-302A × EC-623011 having 6.10, 5.99, 5.85 and 5.66 g 100 seed weight, respectively. CMS-853A × EC-623027 (167.5 cm), P-2–7-1A × EC-601958 (158.75 cm), P-2–7-1A × EC-512682 (164.75 cm), CMS-302A × R-12–96 (162.63 cm), CMS-852A × EC-601751 ( 156.63 cm), CMS- 852A × EC-623023 (161.15 cm) and CMS-302A × EC-623011 (163.5 cm) had reduced plant heights that were significantly lower than the grand mean of LSFH-171 (196.25 cm), DRSH-1 (176.13 cm), and KBSH-53 (189.63 cm). P-2–7-1A × EC-512682 (16.15 cm), and CMS-852A × EC-601751 (15.8 cm) had larger head diameter than the grand mean of LSFH-171 (15.8 cm), DRSH-1 (15.2 cm) and KBSH-53 (15.65 cm). P-2–7-1A × EC-601958 (40.63), CMS-302A × EC-623011 (40.34), CMS-302A × EC-512682 (39.48), and CMS-853A × EC-623027 (39.16) had a greater volume weight (g/100 cc) value than the grand mean of LSFH-171 (36.38), KBSH-53 (38.29). According to the environment means (averages across locations), Bankura had the highest seed yield (2248.62 kg/ ha) and oil yield (852.2 kg /ha), while Baruipur had the lowest seed and oil yields (2,034.4 & 714.2 kg/ha, respectively). This suggested that Bankura possessed the most advantageous climate and that the majority of hybrids were capable of capitalising on it to produce the best seed and oil yields. Across all situations, two national check hybrids, LSFH-171 and KBSH-53, outperformed introduced sunflower hybrids such as P-2–7-1A × EC-601958 (2193.37 kg/ha), and CMS-302A × EC-623011 (2304.62 kg/ha) (Table 2).

### Phenotypic stability

Eight sunflower genotypes growing in four conditions were assessed for seven significant agronomic traits using estimations of phenotypic stability parameters (Table 3). The regression coefficient "b_i_" value deviated significantly from unity (b_i_ >) in sunflower genotypes CMS-302A × EC-623011 (1.051) for seed yield and oil yield (1.037) as well as CMS-853A × EC-623027 (1.137), P-2–7-1A × EC-512682 (1.049), CMS-302A × EC-512682 (1.136), CMS-852A × EC-623023 (1.085) and CMS-302A × EC-623011 (1.051) for seed yeild. Therefore, these sunflower genotypes could be grown under favorable environments. Otherwise, the "b_i_" value was significantly less than unity (b_i_ < 1) in CMS-852A × EC-601751 for seed yield as well as CMS-302A × R-12–96 for plant height. These genotypes are suitable for different environment.

With the exception of genotypes CMS-852A × EC-601751 for volume weight and CMS-302A × EC-623011 for head diameter and volume weight, all sunflower genotypes for all examined features had extremely minor and negligible deviations from regression (S^2^d_i_), which showed significant values. As a result, the genotypes having insignificant values of S^2^d_i_ were more stable. A simultaneous consideration of the three stability parameters, (mean, b_i_ and S^2^d_i_), it can be seen that, the most desired and stable genotypes were CMS-853A × EC-623027, P-2–7-1A × EC-512682, CMS-302A × EC-512682 and CMS-852A × EC-623023 for important agronomic traits.

### Standard heterosis

Across the location hybrid trial (pooled), the highest standard heterosis for seed yield was observed in sunflower hybrid CMS-302A × EC-623011 in which seed yield was recorded 20.90%, 20.91%, 20.95% and 20.90% higher than DRSH-1 at Nimpith, Baruipur, Bankura and PORS(Berhampur) respectively. The significant economic/standard heterosis was also observed in sunflower hybrids viz*.,* CMS-853A × EC-623027 in which seed yield was recorded 19.73% at Bankura, 18.30% at Baruipur, 18.28% at Berhampur higher than DRSH-1. Similarly, in P-2–7-1A × EC-512682 seed yield was recorded more than 17% higher than DRSH-1. In CMS-302A × EC-623011, seed yield was recorded 6.47% higher than KBSH-53 (Table 4).

**Table 4:** Standard/eEconomic heterosis (%) over the national checks (hybrids) for seed yield at different locations of sunflower hybrids

Hybrid	Standard heterosis (%) over the national checks (hybrids) for seed yield (kg/ha)											
	Nimpith			Baruipur			Bankura			Berhampur		
	LSFH-171	DRSH-1	KBSH-53	LSFH-171	DRSH-1	KBSH-53	LSFH-171	DRSH-1	KBSH-53	LSFH-171	DRSH-1	KBSH-53
CMS-853A × EC-623027	-2.98	18.3^b^	4.19	-3.01	18.30^b^	4.16	-1.16	19.73^b^	5.14^a^	-3.00	18.28^b^	4.13
P-2–7-1A × EC-601958	-5.44^a^	15.3^b^	1.56	-5.94^a^	14.72^b^	1.01	-4.62	15.53^b^	1.45	-5.94^a^	14.69^b^	0.98
P-2–7-1A × EC-512682	-3.42	17.8^b^	3.72	-3.44	17.77^b^	3.70	-2.13	18.55^b^	4.11	-3.42	17.77^b^	3.69
CMS-302A × R-12–96	-7.98^a^	12.2^b^	-1.17	-7.99^a^	12.23^b^	-1.18	-7.43^a^	12.13^b^	-1.54	-7.96^a^	12.23^b^	-1.19
CMS-852A × EC-601751	-12.03^a^	7.3^a^	-5.53^a^	-12.05^b^	7.28*	-5.54*	-11.19^b^	7.58^a^	-5.53^a^	-12.05^b^	7.24^a^	-5.58^a^
CMS-302A × EC-512682	-8.86^a^	11.1^b^	-2.12	-8.88^a^	11.14^b^	-2.14	-8.19^a^	11.20^b^	-2.35	-8.88^a^	11.11^b^	-2.17
CMS-852A × EC-623023	-5.90^a^	14.8^b^	1.06	-5.92^a^	14.75^b^	1.03	-5.22^a^	14.80^b^	0.81	-5.90^a^	14.75^b^	1.03
CMS-302A × EC-623011	-0.86	20.9^b^	6.47^a^	-0.87	20.91^b^	6.46*	-0.14	20.95^b^	6.21	-0.85	20.90^b^	6.45^a^

^a,^^b^: Standard heterosis over national check varities exceeding 5 and 10%, respectively

Similarly, in hybrid trial (pooled), the highest standard heterosis for oil yield was observed in CMS-302A × EC-623011 in which oil yield was recorded more than 14% higher than all national check varieties at different locations. The significant economic/standard heterosis over national check varities exceeding 10% was also observed in sunflower hybrids viz*.,* CMS-853A × EC-623027, P-2–7-1A × EC-601958, and P-2–7-1A × EC512682. Similarly, in CMS- 852A × EC-623023, oil yield was recorded more than 5% higher over all three national check varieties (Table 5).

**Table 5:** Standard/economic heterosis (%) over the national checks (hybrids) for oil yield (kg/ha) at different locations of sunflower hybrids

Hybrid	Standard heterosis (%) over the national checks (hybrids) for oil yield (kg/ha)											
	Nimpith			Baruipur			Bankura			Berhampur		
	LSFH-171	DRSH-1	KBSH-53	LSFH-171	DRSH-1	KBSH-53	LSFH-171	DRSH-1	KBSH-53	LSFH-171	DRSH-1	KBSH-53
CMS-853A × EC-623027	12.34^b^	14.42^b^	13.15^b^	12.48^b^	14.49^b^	13.04^b^	14.45^b^	15.76^b^	13.97^b^	12.49^b^	14.41^b^	12.95^b^
P-2–7-1A × EC601958	10.09^b^	12.13^b^	10.88^b^	9.40^a^	11.36^b^	9.95*	10.75^b^	12.02^b^	10.28^b^	9.38^a^	11.25^b^	9.83^a^
P-2–7-1A × EC512682	12.73^b^	14.82^b^	13.54^b^	12.92^b^	14.94^b^	13.48^b^	14.24^b^	15.55^b^	13.76^b^	12.90^b^	14.82^b^	13.36^b^
CMS-302A × R-12–96	0.31	2.17	1.03	0.41	2.21	0.91	0.88	2.03	0.45	0.51	2.23	0.92
CMS-852A × EC-601751	-5.86^a^	-4.12	-5.19^a^	-5.69^a^	-4.00	-5.22^a^	-4.81	-3.72	-5.21^a^	-5.65^a^	-4.04	-5.26^a^
CMS-302A × EC-512682	4.17	6.11^a^	4.92	4.28	6.15^b^	4.80	4.97	6.17*	4.52	4.36	6.15^a^	4.79
CMS-852A × EC-623023	6.03^a^	8.00^a^	6.80^a^	6.10^a^	8.00^a^	6.63^a^	6.86a	8.08*	6.41^a^	6.29^a^	8.10^a^	6.72^a^
CMS-302A × EC-623011	14.33^b^	16.45^b^	15.15^b^	14.49^b^	16.54^b^	15.06^b^	15.17^b^	16.48**	14.68^b^	14.53^b^	16.49^b^	15.00^b^

^a, b^ Standard heterosis over national check varities exceeding5 and 10%, respectively

## Discussion

Sunflower yield is a cumulative function of numerous components. The yield is a complex expression of a large number of genes engaged in the physiochemical activities of the plant system. Sunflower hybrids enable the composition and balance of one or two components to be optimised, resulting in a high yield. Sunflowers are mostly cultivated for their oil. However, because there is no mechanism for determining the oil content of sunflower seeds, all sunflower producers choose high-yielding sunflower cultivars/hybrids over those with a higher oil yield. Due to their increased height, certain sunflower hybrids also experienced lodging problems. Thus, intelligent selection may be employed to get a high yield in these cases [21]. Experimental results in this study indicate that sunflower hybrids exhibit variances in yield and yield contributing factors. For the many characters sensitive to environmental variations, the (GE) interaction lowers association between phenotypic and genotypic values and contributes to bias in estimates of gene effects; such traits are less amenable to selection. To reduce the consequences of GE and increase the accuracy and refinement of genotype selection, yield and stability of performance should be taken into account simultaneously [22].

A pooled analysis of variance for the genotype × environment interaction revealed very significant differences between hybrids, indicating the presence of substantial genetic diversity. Significant differences in habitats suggested that the hybrid had been examined over a period of several seasons. These findings are consistent with those of Singamsetti, Shahi [23].

The regression analysis proposed by Eberhart and Russell [15] was used to estimate regression coefficient (b_i_) and the deviations from regression (S^2^d_i_). The regression coefficient (b_i_) shows the response of a genotype to varying environments, while S^2^d_i_ measures the dispersion around the regression line. Genotype with (b_i_) value not significantly different from unity and S^2^d_i_ not significantly from zero or small as possible is considered as stable genotype. A stable genotype will be more desirable when it has a mean yield greater than the average yield of all genotypes. In this research, regression coefficients of newly bred hybrids ranged from 0.95 (CMS-852A × EC-601751) to 1.11 (CMS-853A × EC-623027) for seed yield. This variation in regression coefficients indicated that sunflower genotypes have different responses to environmental changes. Similarly, Akcura, Kaya [24] found that the regression coefficient for genotypes was considerably higher than unity, indicating that their seed yields were above the grand mean. These genotypes are sensitive to environmental changes and would be recommended for cultivation under ideal environments only.

Estimates of the regression coefficient and the deviations from the regression indicated that each character had a wide range of values seed yield (b_i_: 0.571 to 1.137, S^2^d_i_: 7.75 to 830.91); oil content (b_i_: 0.871 to 1.209, S^2^d_i_: 0.00023 to 0.516); oil yield (b_i_: 0.776 to 1.135, S^2^d_i_: 5.806 to 166.42); 100 seed weight (b_i_ i: 0.871 to 1.209, S^2^d_i_: 0.000227 to 0.1637); plant height (b_i_: 0.574 to 1.478, S^2^d_i_: 3.052 to 17.739); head diameter (b_i_: 0.648 to 1.381, S^2^d_i_: 0.00349 to 0.0611); volume weight (b_i_: 0.529 to 1.248, S^2^d_i_: 0.0996 to 0.9335). Three characteristics were used to assess the genotype's phenotypic stability: mean values, regression coefficient, and deviations from regression. A stable genotype should have a high mean performance, unit regression coefficient, and the smallest feasible deviations from regression [15]. In this experiment, eight superior hybrids were evaluated at four locations by comparing three national checks. Hybrids were classified into three groups based on their stability parameters: Group I: high mean with regression coefficient values near unity and non-significant to zero deviations from regression, stable, and adaptable to all situations P-2–7-1A × EC-601958 and CMS-302A × R-12–96. Group II: high mean with a b_i_ significant and greater than unity and a non-significant S^2^d_i_, which is ideal for favourable settings CMS-853A × EC-623027, P-2–7-1A × EC-512682. Group III: high mean with b_i_ significant and less than unity and S^2^d_i_, ideal for difficult settings CMS-302A × EC-512682. Sheoran, Amit [25] and Ghaffari, Gholizadeh [26] reported more adaptive and stable hybrids with a high mean, a regression coefficient (b_i_) near unity, and deviations from the regression (S^2^d_i_) near zero on seed yield. Tyagi, Dhillon [27] reported stable sunflower hybrids for seed yield; Tabrizi, Hassanzadeh [28] reported stable hybrids for oil yield; Neelima and Parameshwarappa [29] reported stable hybrids for head diameter, number of filled seeds per head, 100 seed weight, and seed yield per plant. By and large, hybrids that were shown to be stable for seed yield also demonstrated stability for one or more yield component traits. This revealed that the stability of various component features might account for the observed stability of diverse hybrids in terms of seed yield. By prioritising stability in specific components, the odds of picking a stable hybrid can be increased. CMS-302A × EC-623011, CMS-853A × EC-623027, and P-2–7-1A × EC-512682 are the most stable hybrids for seed, oil yields, with a reasonable balance of plant height and volume weight, because their regression coefficients were close to one (b_i_ = 1) and had the lowest deviations from regression (S^2^d_i_), respectively. It was found by Hayward and Lawrence [30] that the regression parameter measuring the response to environment is highly heritable and influenced by genes with additive effects. Furthermore, S^2^d values appeared to be the most reliable indicator of stability [31].

Similarly, the aforementioned high heterotic hybrids exhibited desirable considerable heterosis for component characteristics like seed and oil output. Heterosis research revealed that the direction and degree of heterosis varied between hybrids and environments. CMS-302A × EC-623011, P-2–7-1A × EC-512682, CMS-853A × EC-623027, and P-2–7-1A × EC-601958 all achieved considerably higher oil yield values as compared to national control hybrids LSFH-171, DRSH-1, and KBSH-53 at all four locations. Other hybrids, such as CMS-852A × EC-623023, yielded much more oil than DRSH-1 in all four locations and significantly more oil than KBSH-53 and LSFH-171 in two locations, Nimpith and Baruipur. Other resechers [32–37] also reported standard (economic) heterosis of sunflower hybrids for seed yield and oil yield.

## Conclusion

The purpose of the economic heterosis estimation in this study was to find the optimal combination of parents with a high degree of usable heterosis for seed yield and other yield attributing characteristics for their prospects for future usage in sunflower breeding programmes. The study concluded that CMS-302A × EC-623011, P-2–7-1A × EC-512682 and CMS-853A × EC-623027 exhibited great seed yield and stability in terms of seed production, oil yield, head diameter, volume weight, and oil content. The availability of cytoplasmic male sterile (CMS) lines and hybrid seed production technology will enable commercialization of these crosses following rigorous evaluation in multi-location trials and an all-India trial to determine their superiority across locations, years, and soil types for commercial utility.

## Supplementary Information

**Additional file 1: Supplementary file 1.** List of genotypes and its important traits.

**Additional file 2:**  **Supplementary file 2.** Metrological Data at different location during 2017-18.

**Additional file 3:** **Supplementary file 3.** Metrological Data at different location during 2018-19.

**Additional file 4: Supplementary fig. 2.** Glimpses of field performance of some newly bred sunflower hybrids at various agro-ecological zones during stability analysis.

## Data Availability

All the data is available in the submitted manuscript.
