# Supplementary material for: Yield stability and economic heterosis analysis in newly bred sunflower hybrids throughout diverse agro-ecological zones
Source: BMC Plant Biol. 2022 Dec 12;22:579. doi: 10.1186/s12870-022-03983-1 (PMC9743611; doi:10.1186/s12870-022-03983-1)
Supplement: Supplementary file 1 — Additional file 1: Supplementary file 1. List of genotypes and its important traits. [file 12870_2022_3983_MOESM1_ESM.docx]

Supplementary file: 1. **List of genotypes and its important traits**

| **Parents** | **Origin** | **Important traits** |
| --- | --- | --- |
| CMS lines | | |
| P-89-1A, CMS-207A, CMS-302A, | IIOR, Hyderabad | High seed yield. |
| CMS-10A | IIOR, Hyderabad | Early maturity and high oil content |
| P-2-7-1A | IIOR, Hyderabad | High Test weight and high seed yield |
| CMS-16A, CMS-850A, CMS-852A, CMS-853A | ORS, Latur | Early maturity, Dwarf and high oil content |
| CMS-103A, CMS-107A, | UAS, Bangalore | Early maturity and high oil content |
| Restorer lines | | |
| R-138-2, R-630, R-104, R-12-96 | UAS, Bangalore | High seed yield and black colour seed. |
| R-1-1 | IIOR, Hyderabad | High Test weight and high seed yield |
| R-104, R-107 | UAS, Bangalore | High seed yield and black colour seed. |
| EC-602060, EC-601978, R-341 | ORS, Latur | Early maturity, high Test weight & black colour seed. |
| EC-623027(M) | ORS, Latur | High test weight and high seed yield |
| EC-623021, EC-623023, | ORS, Latur | High Test weight and high seed yield |
| EC-601751, EC-601725 | ORS, Latur | Early maturity, high Test weight & black colour seed. |
| EC-623029, EC-601958, EC-623011 | ORS, Latur | High oil content, stress tolerant, black colour seed |
| EC-623016, EC-512682 | ORS, Latur | Early maturity, Downy mildew and stress resistant, |
| R-6D-1 | UAS, Bangalore | High oil content. |
