# Supplementary material for: Yield stability and economic heterosis analysis in newly bred sunflower hybrids throughout diverse agro-ecological zones
Source: BMC Plant Biol. 2022 Dec 12;22:579. doi: 10.1186/s12870-022-03983-1 (PMC9743611; doi:10.1186/s12870-022-03983-1)
Supplement: Supplementary file 2 — Additional file 2: Supplementary file 2. Metrological Data at different location during 2017-18. [file 12870_2022_3983_MOESM2_ESM.docx]

Supplementary file 2: Metrological Data at different location during 2017-18

| Locations | December | | | January | | | February | | | March | | | April | | |
| --- | --- | --- | --- | --- | --- | --- | --- | --- | --- | --- | --- | --- | --- | --- | --- |
|  | Tempature  (Average)  High/low | Humidity  average | Rain | Tempature  (Average)  High/low | Humidity  average | Rain | Tempature  (Average)  High/low | Humidity  average | Rain | Tempature  (Average)  High/low | Humidity  average | Rain | Tempature  (Average)  High/low | Humidity  average | Rain |
| Nimpith | 25^0^/14^0^ | 70% | 1 | 24^0^/15^0^ | 74% | 2 | 27^0^/14^0^ | 60% | 0 | 32^0^/17^0^ | 73% | 1 | 36^0^/27^0^ | 70% | 1 |
| Baruipur | 27^0^/13^0^ | 44% | 0 | 26^0^/12^0^ | 46% | 1 | 29^0^/16^0^ | 45% | 1 | 29^0^/18^0^ | 68% | 1 | 35^0^/23^0^ | 68% | 2 |
| Bankura | 27^0^/13^0^ | 42% | 0 | 27^0^/12^0^ | 42% | 0 | 31^0^/15^0^ | 43% | 0 | 34^0^/25^0^ | 65% | 2 | 38^0^/30^0^ | 62% | 2 |
| PORS(Berhampur) | 15^0^/12^0^ | 45% | 0 | 15^0^/10^0^ | 40% | 0 | 20^0^/15^0^ | 40% | 0 | 25^0^/17^0^ | 55% | 0 | 27^0^/22^0^ | 60% | 1 |
