# Supplementary material for: Yield stability and economic heterosis analysis in newly bred sunflower hybrids throughout diverse agro-ecological zones
Source: BMC Plant Biol. 2022 Dec 12;22:579. doi: 10.1186/s12870-022-03983-1 (PMC9743611; doi:10.1186/s12870-022-03983-1)
Supplement: Supplementary file 3 — Additional file 3: Supplementary file 3. Metrological Data at different location during 2018-19. [file 12870_2022_3983_MOESM3_ESM.docx]

Supplementary file 3: Metrological Data at different location during 2018-19

| Locations | December | | | January | | | February | | | March | | | April | | |
| --- | --- | --- | --- | --- | --- | --- | --- | --- | --- | --- | --- | --- | --- | --- | --- |
|  | Tempature  (Average)  High/low | Humidity  average | Rain | Tempature  (Average)  High/low | Humidity  average | Rain | Tempature  (Average)  High/low | Humidity  average | Rain | Tempature  (Average)  High/low | Humidity  average | Rain | Tempature  (Average)  High/low | Humidity  average | Rain |
| Nimpith | 23^0^/13^0^ | 72% | 1 | 26^0^/15^0^ | 64% | 1 | 27^0^/16^0^ | 56% | 0 | 32^0^/17^0^ | 69% | 1 | 36^0^/27^0^ | 65% | 1 |
| Baruipur | 27^0^/15^0^ | 48% | 0 | 26^0^/11^0^ | 66% | 2 | 29^0^/16^0^ | 65% | 2 | 29^0^/18^0^ | 58% | 0 | 35^0^/23^0^ | 62% | 1 |
| Bankura | 29^0^/13^0^ | 52% | 0 | 23^0^/12^0^ | 48% | 0 | 31^0^/15^0^ | 49% | 0 | 34^0^/25^0^ | 64% | 2 | 38^0^/30^0^ | 66% | 2 |
| PORS(Berhampur) | 18^0^/12^0^ | 65% | 1 | 15^0^/10^0^ | 45% | 0 | 20^0^/15^0^ | 55% | 0 | 25^0^/17^0^ | 65% | 2 | 27^0^/22^0^ | 50% | 0 |
