# Supplementary material for: Yield stability and economic heterosis analysis in newly bred sunflower hybrids throughout diverse agro-ecological zones
Source: BMC Plant Biol. 2022 Dec 12;22:579. doi: 10.1186/s12870-022-03983-1 (PMC9743611; doi:10.1186/s12870-022-03983-1)
Supplement: Supplementary file 4 — Additional file 4: Supplementary fig. 2. Glimpses of field performance of some newly bred sunflower hybrids at various agro-ecological zones during stability analysis. [file 12870_2022_3983_MOESM4_ESM.pdf]

Supplementary fig. 2 Glimpses of field performance of some newly bred sunflower hybrids at various agro-ecological zones during stability analysis

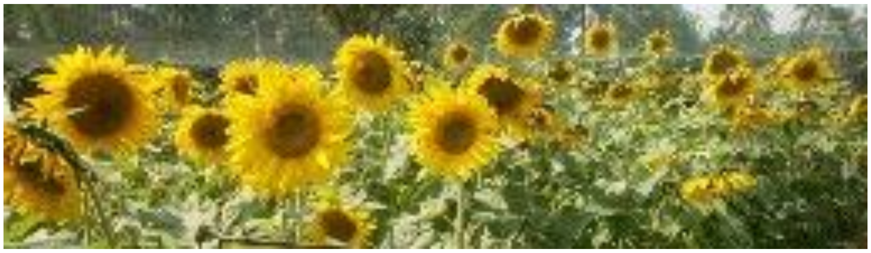

Field view: F_1 (CMS-853A × EC-623027) at PORS, Berhampur, Murshidabad, West Bengal, India

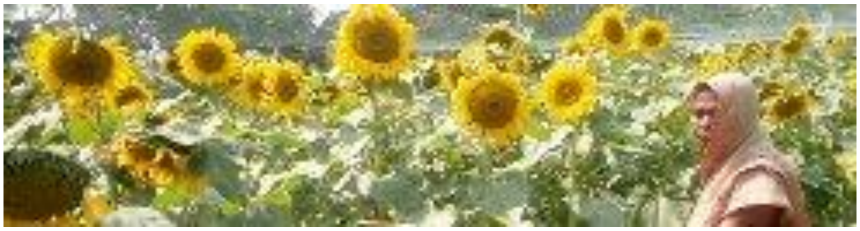

Field view: F_1 (CMS-853A X EC-623027) at Nimpith, South 24 Parganas, West Bengal, India

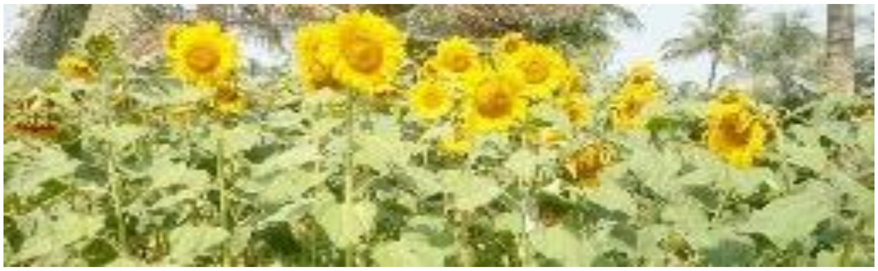

Field view: F1 (CMS-302A × EC-623011) at Bankura, West Bengal, India

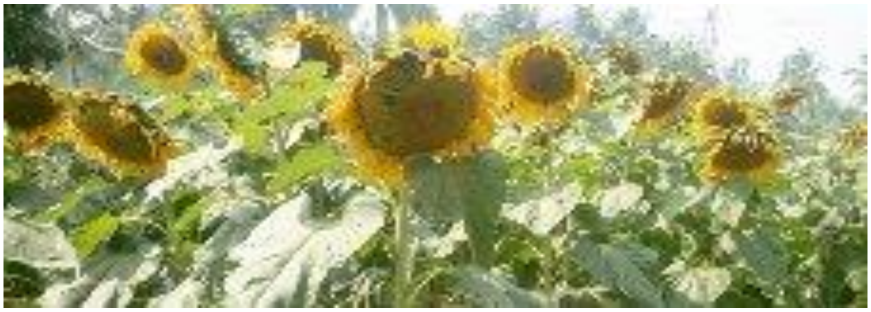

Field view: F_1 (CMS-302A X R-12-96) at Nimpith, South 24 Parganas, West Bengal, India

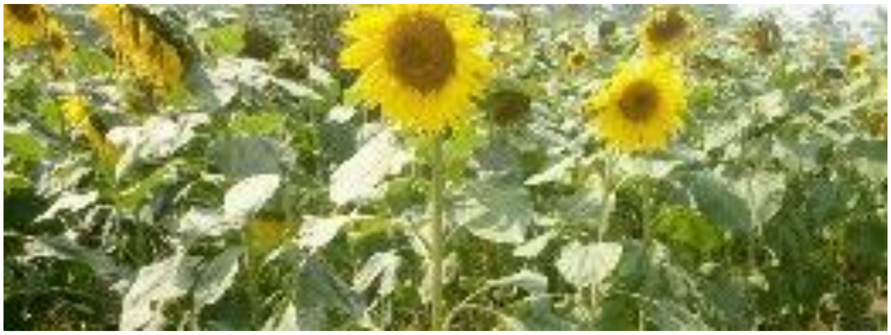

Field view: F_1 (CMS-853A X EC-623027) at Baruipur, South 24 Parganas, West Bengal, India
